# Appendectomy Hospital Stay: No Difference in Obese Adult or Pediatric Patient Length of Stay Compared to Nonobese Patients

**DOI:** 10.31486/toj.19.0116

**Published:** 2021

**Authors:** Eric Lorio, David H. Ballard, Elizabeth Guarisco, James Hughes, Forrest D. Griffen, Navdeep S. Samra

**Affiliations:** ^1^ Department of Internal Medicine, University of Texas Health Science Center at San Antonio, San Antonio, TX; ^2^ Department of Radiology, Mallinckrodt Institute of Radiology, Washington University School of Medicine, St. Louis, MO; ^3^ Department of Anesthesiology, West Virginia University, Morgantown, WV; ^4^ Department of Surgery, Louisiana State University School of Medicine–Shreveport, Shreveport, LA

**Keywords:** *Appendectomy*, *body mass index*, *inpatients*, *length of stay*, *obesity*, *postoperative complications*

## Abstract

**Background:** Studies of adult and pediatric patients undergoing appendectomy have reported variable outcomes and operative metrics related to the effect of obesity. The purpose of this study was to investigate the effect of obesity in adult and pediatric patients undergoing appendectomy at our institution.

**Methods:** This single-center retrospective study evaluated the relationship between length of hospital stay for appendectomy and body mass index (BMI). Data obtained from the electronic medical record included age, sex, weight, height, BMI, the number of hours the patient experienced symptoms prior to presentation to the emergency room, the number of hours the patient was admitted prior to surgery, the number of hours of hospital admission after surgery, perforated appendix, preoperative comorbidities, and evidence of preoperative sepsis.

**Results:** During the 3-year study period, 118 adults and 38 children who underwent appendectomy composed the study groups. Patients were stratified by obese and nonobese, with obesity defined as BMI ≥30.0 kg/m^2^. In adults, we found no significant difference between length of stay in obese (n=45) and nonobese (n=73) patients (79.6 ± 65.5 hours vs 101.6 ± 123.0 hours; *P*=0.21). In children, we found no significant difference between length of stay in obese (n=9) and nonobese (n=29) patients (92.9 ± 64.6 hours vs 109.0 ± 93.5 hours; *P*=0.54).

**Conclusion:** Obesity did not affect length of stay in adults and children who underwent appendectomy in the present series.

## INTRODUCTION

Appendectomy is one of the most common surgical procedures worldwide, whether performed for management of acute appendicitis or as an addition to a larger abdominal surgery.^1^ In the United States, approximately 200,000 appendectomies are performed annually.^1^ As such, patients undergoing appendectomy represent a sizable proportion of the general surgery patient population, and obesity may be an important factor in determining patient morbidity related to appendectomy performed for management of acute appendicitis.

Albeit not specific to appendectomy, studies suggest that high body mass index (BMI) is positively associated with in-hospital mortality and increased length of stay (LOS).^2,3^ Akinyemiju et al concluded that higher BMI was associated with increased risk of mortality and longer hospital stay in a cohort of more than 800,000 patients admitted for various medical and cancer-related diagnoses.^2^ Similarly, Lewis et al found that obesity was associated with increased comorbid illness and with significantly longer intensive care unit and hospital LOS.^3^ However, in a cohort of 272 patients who underwent appendectomy, no significant differences were found in postoperative recovery, appendix perforation status, or mortality between obese and nonobese patients, and Towfigh et al recommended no change in appendicitis management for obese patients.^4^ A study of a large national pediatric database that compared patients who underwent appendectomy to patients undergoing other intestinal operations showed that obese appendectomy pediatric patients had significantly longer hospital LOS compared nonobese patients, but no significant difference in LOS was seen between obese and nonobese patients undergoing other intestinal operations.^5^ Other studies have reported worse outcomes in obese adults and longer operating times in obese adults and children.^6-8^ The purpose of this study was to investigate the effect of obesity in adult and pediatric patients undergoing appendectomy at our institution.

## METHODS

This study is a retrospective analysis of adult and pediatric populations who had an appendectomy at our institution during a 3-year period. Review of electronic medical records was conducted after obtaining institutional review board approval. Obesity was defined as BMI ≥30.0 kg/m^2^. Data obtained from the electronic medical record included age, sex, weight, height, BMI, the number of hours the patient experienced symptoms prior to presentation to the emergency room, the number of hours the patient was admitted prior to surgery, and the number of hours of hospital admission after surgery. *International Classification of Diseases, Tenth Revision* codes were used to identify perforation status of the appendix. All cases were also reviewed for evidence of appendix perforation by direct intraoperative visualization, preoperative comorbidities, and evidence of preoperative sepsis. Sepsis was defined as having at least 2 of the following in the presence of possible infection: central body temperature >101 °F (38.3 °C) or <96.8 °F (36 °C), heart rate ≥90 bpm, or respiratory rate ≥20 breaths per minute.^9^ For comorbidities, each condition was recorded, and a comorbidity score (0, 1, or ≥2) was created as the sum of the number of conditions per patient. BMI was stratified, and LOS was then compared between obese patients and nonobese patients.

A secondary analysis assessed whether preoperative admission time had an effect on postoperative admission time in the obese patient vs the nonobese patient. The purpose of this analysis was to determine if obese patients required more preoperative admission time to optimize for surgery. Obese patients were separated into groups: <24 hours preoperative admission time and ≥24 hours preoperative admission time. Nonobese patients were separated using the same categories. Patients were also grouped according to number of comorbidities (0, 1, or ≥2). Postoperative LOS was subsequently compared.

Fisher exact test and *z* score were used to calculate statistical values. Multivariable analysis was performed secondarily, controlling for the potential confounders of BMI, sex, age, and number of comorbidities. *P* values <0.05 were considered statistically significant.

## RESULTS

The Figure shows the comparison in LOS and related factors for all adult and pediatric patients.

**Figure.**
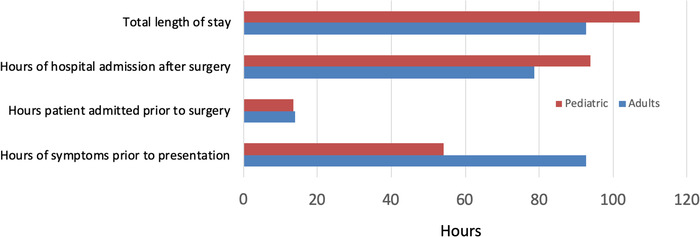
**Length of stay factors including hours admitted after surgery, hours admitted prior to surgery, duration of symptoms prior to hospital arrival, and overall length of stay in the adult patients compared to the pediatric patients in the study cohort. Upper bars show pediatric data; lower bars show adult data.**

### Adult Patients

We identified 118 adult patients who underwent appendectomy (Table 1). In the adult population, 58% were male, 42% were female, and their mean age was 38 years. Seventy-three patients (62%) had a BMI <30 kg/m^2^, while 45 patients (38%) had a BMI ≥30 kg/m^2^. A slight majority of female patients were obese (n=26; 52%), whereas only 28% (n=19) of male patients were obese (*P*=0.012). Obese patients had a similar appendix perforation rate (18%) compared to nonobese patients (14%) (*P*=0.700). We found no significant difference in the number of comorbidities between obese and nonobese patients (*P*=0.42). Overall, 45 patients (38%) had no comorbidities, 35 patients (30%) had 1 comorbidity, and 38 patients (32%) had 2 or more comorbidities.

**Table 1.** Adult Patient Characteristics, Overall and by Body Mass Index (BMI) Classification

Variable	All Patients, n=118	Nonobese, BMI <30 kg/m^2^, n=73	Obese, BMI ≥30 kg/m^2^, n=45	*P* Value
Age, years, mean	38	37	40	0.36
Sex				0.012
Female^a^	50	24 (48)	26 (52)	
Male^a^	68	49 (72)	19 (28)	
Perforated appendix	18 (15)	10 (14)	8 (18)	0.700
Comorbidities		1.2 (mean)	1.2 (mean)	0.42
0	45 (38)	26 (36)	19 (42)	
1	35 (30)	25 (34)	10 (22)	
≥2	38 (32)	22 (30)	16 (36)	
Hours patient experienced symptoms prior to presentation, mean	92.6	39.3	54.1	0.26
Hours patient admitted prior to surgery, mean	14.0	15.8	11.2	0.19
Hours of hospital admission after surgery, mean	78.6	85.5	67.5	0.28

^a^Percentages are calculated across the row and not by column.

Note: Data are presented as n (%) unless otherwise indicated.

We found no significant difference in length of time of symptoms before presentation to the emergency department, length of time patients were admitted prior to their surgery, or length of time patients were in the hospital after surgery was performed (Table 1).

LOS in adult patients was not affected by obesity at our institution (Table 2). In multivariate analysis, controlling for BMI, sex, age, and number of comorbidities, LOS in obese adult patients (79.6 ± 65.5 hours) was not significantly different compared to nonobese adult patients (101.6 ± 123.0 hours) (*P*=0.21). In multivariate analysis, none of the following was a significant predictor: the length of time of symptoms prior to emergency department presentation, preoperative admitted time, and postoperative admitted time (*P*≥0.2 in all comparisons).

**Table 2.** Hospital Length of Stay (LOS) in Adult (n=118) and Pediatric (n=38) Patients by Body Mass Index (BMI) Classification

	Nonobese, BMI <30 kg/m^2^	Obese, BMI ≥30 kg/m^2^	*P* Value
Adult LOS, hours, mean ± SD	101.6 ± 123.0	79.6 ± 65.5	0.21
Pediatric LOS, hours, mean ± SD	109.0 ± 93.5	92.9 ± 64.6	0.54

When comparing patients receiving <24 hours of inpatient care preoperatively with patients receiving ≥24 hours preoperatively, we found no significant change in the postoperative duration of inpatient admission for obese vs nonobese patients regardless of the number of comorbidities (*P*>0.05 in all comparisons). Comparing <24 hours vs ≥24 hours preoperatively, we found no significant difference in proportion of perforated appendix, sepsis, or mean number of comorbidities (*P*>0.05 in all comparisons) between obese and nonobese patients. However, duration of inpatient admission preoperatively was 11.3 hours longer for patients with one or more comorbidities compared to patients without a comorbidity when controlling for BMI, sex, and age (*P*=0.015).

### Pediatric Patients

We identified 38 pediatric patients who had appendectomies during the study period, 23 males (61%) and 15 females (39%) with a mean age of 9 years (Table 3). Nine patients (24%) were obese. Most pediatric patients had no comorbidities (76%). A greater percentage of the pediatric patients had a perforated appendix (26%) compared to adults (15%).

**Table 3.** Pediatric Patient Characteristics, Overall and by Body Mass Index (BMI) Classification

Variable	All Patients, n=38	Nonobese, BMI <30 kg/m^2^, n=29	Obese, BMI ≥30 kg/m^2^, n=9	*P* Value
Age, years, mean	9	9	11	0.40
Sex				0.26
Female^a^	15	10 (67)	5 (33)	
Male^a^	23	19 (83)	4 (17)	
Perforated appendix	10 (26)	9 (31)	1 (11)	0.25
Comorbidities		0.11 (mean)	0.2 (mean)	0.45
0	29 (76)	21 (72)	8 (89)	
1	4 (11)	3 (10)	1 (11)	
≥2	5 (13)	5 (17)	0	
Hours patient experienced symptoms prior to presentation, mean	54.2	62.3	28.1	0.015
Hours patient admitted prior to surgery, mean	13.5	15.0	8.8	0.25
Hours of hospital admission after surgery, mean	93.8	95.5	86.9	0.78

^a^Percentages are calculated across the row and not by column.

Note: Data are presented as n (%) unless otherwise indicated.

We observed a significant difference in the duration of experiencing symptoms before presentation to the emergency department (*P*=0.015), with the obese population presenting after a mean of 28.1 hours of symptoms and the nonobese population presenting at a mean of 62.3 hours. We found no significant difference in the number of hours a pediatric patient spent admitted preoperatively (*P*=0.25) or postoperatively (*P*=0.78) between obese and nonobese patients. We also noted no difference in the number of comorbidities (*P*=0.45) between obese and nonobese pediatric patients. Obese patients were found to have a similar perforation rate when compared to nonobese patients (*P*=0.25).

We found no significant difference in the total LOS between obese and nonobese pediatric patients (*P*=0.54) (Table 2). Stratifying all patients—obese and nonobese—who received <24 hours vs ≥24 hours of inpatient care preoperatively, we found no significant difference in the proportion of perforated appendix, sepsis, or mean number of comorbidities (*P*>0.05 in all comparisons).

## DISCUSSION

In this study, we found no difference in LOS between obese and nonobese patients receiving appendectomy in either the adult or pediatric subgroup. Secondary observations included (1) a significantly higher incidence of obese adult female patients vs obese adult male patients (*P*=0.012), (2) an increased length of inpatient hospitalization preoperatively in adult patients with comorbidities when controlling for all variables (*P*=0.015), and (3) a significant difference between the obese and the nonobese pediatric patients in the duration of experiencing symptoms before presentation to the emergency department (*P*=0.015).

The high proportion of adult obese female patients in part may reflect the rising incidence of female obesity in the United States.^10^ Although Ballard et al did not investigate appendectomy, their surgical study reported a higher incidence of obese female patients than male patients.^11^

Contrary to our findings, Garey et al^6^ and Knott et al^7^ demonstrated associations of obesity with longer LOS after appendectomy. Those studies also reported longer operative times, increased postoperative analgesics, and worse outcomes in obese appendectomy patients.

Regarding pediatric patients undergoing appendectomy, obesity has been associated with longer surgeries, longer hospital stays, and increased postoperative infections when compared to nonobese children.^12,13^ However, we found no significant increase in LOS in our pediatric population because of obesity, a finding that agrees with Blanco et al who found that obesity in general did not contribute to increased LOS or postoperative infections in a pediatric population.^14^ Michailidou et al showed significantly longer operative times in obese pediatric patients receiving appendectomy vs nonobese pediatric patients.^8^ However, that study showed no increase in pediatric postoperative complication rate after 30 days and concluded that obesity is not an independent risk factor for postoperative complications following laparoscopic appendectomy.

Our study had several limitations, including its small sample size and retrospective nature. LOS and related metrics were the primary variables of this investigation, and the time durations obtained from electronic medical record charting are prone to administrative and charting errors in the hours of time spent in the hospital. BMI is an imperfect tool for determination of obesity because it does not evaluate body type. We used a BMI ≥30 kg/m^2^ to define the obese group. The study of subsets such as overweight (BMI 25-29 kg/m^2^), obese (≥30 kg/m^2^), severely obese (≥35 kg/m^2^), and morbidly obese (≥40 kg/m^2^) was not practical given our small sample size. The reason for the significantly longer admission time for adult patients with comorbidities is unclear. Contributing factors may have included delayed diagnosis of appendicitis given comorbidities confounding the clinical presentation, preoperative optimization, or both; however, these factors were not objectively assessed. Although others have reported obesity-related longer operative times and higher rates of postoperative infections,^12,13^ we did not assess for obesity-related longer operative times, which is a limitation.

## CONCLUSION

This study adds support to the body of literature indicating that obesity is not a risk factor for a prolonged LOS after appendectomy. We noted no apparent change in the postoperative LOS in patients who spent lengthened inpatient time preoperatively, suggesting that prolonged overall LOS can be mitigated by adequate optimization before surgery in at-risk patients.

## Notes

This article meets the Accreditation Council for Graduate Medical Education and the American Board of Medical Specialties Maintenance of Certification competencies for Patient Care and Medical Knowledge.
